# Reduction of heterozygosity (ROH) as a method to detect mosaic structural variation

**DOI:** 10.1111/pbi.12691

**Published:** 2017-03-16

**Authors:** Fabio Marroni, Davide Scaglione, Sara Pinosio, Alberto Policriti, Mara Miculan, Gabriele Di Gaspero, Michele Morgante

**Affiliations:** ^1^ Dipartimento di Scienze agroalimentari, ambientali e animali Università di Udine Udine Italy; ^2^ Istituto di Genomica Applicata (IGA) Udine Italy; ^3^ IGA Technology Services Udine Italy; ^4^ Parco Tecnologico Padano Lodi Italy; ^5^ Institute of Biosciences and Bioresources National Research Council Sesto Fiorentino (Firenze) Italy; ^6^ Department of Mathematics and Computer Science University of Udine Udine Italy; ^7^ Present address: Dipartimento di Scienze agroalimentari, ambientali e animali Università di Udine Via delle Scienze 206 Udine 33100 Italy

**Keywords:** mosaic structural variation, clonal variation, reduction of heterozygosity, next‐generation sequencing, SNPs, *Vitis vinifera*

Structural variation (SV) is determined by genome rearrangements that affect large (>1 kb) segments of DNA such as inversions and balanced translocations (balanced SV) or insertions, deletions and duplications (unbalanced SV) (Feuk *et al*., 2006). SV occurred in somatic cells originating from the same fertilized egg and affecting only a portion of the cells of an organism give rise to mosaic structural variation (King *et al*., 2015). Mosaicism is relevant in fruit tree breeding, where valuable seedlings are immortalized by vegetative propagation. Grapevine (*Vitis vinifera*) is a vegetatively propagated crop in which mosaicism contributes to phenotypic differences within cultivars. One example is Pinot noir, a black‐berried cultivar that gave rise to several clones harbouring somatic and/or mosaic variation such as Pinot blanc, Pinot gris and Pinot Meunier.

The absence of pigmentation in Pinot blanc has been attributed to a heterozygous deletion affecting a region of chromosome 2 involved in anthocyanin synthesis (Yakushiji *et al*., 2006). Pinot gris has grayish berries due to a localized synthesis of anthocyanins in epidermal cells. The causal mutation was originally identified as a deletion occurring only in the L2 layer of chromosome 2, which removed the functional alleles of two adjacent *MybA* genes (Vezzulli *et al*., 2012). Recent experiments confirmed the mosaic nature of the event, but suggested that the causal mutation is a large balanced SV consisting in the replacement of the chromosomal region carrying the red haplotype with the corresponding region carrying the white haplotype (Pelsy *et al*., 2015). Currently, next‐generation sequencing (NGS) is the technology of choice for the detection of SV. Available methods are based on Paired End Mapping (PEM) or Depth of Coverage (DOC) (Marroni *et al*., 2014; Medvedev *et al*., 2009), but these approaches are not ideal for the detection of mosaic SV. PEM signatures reside in the flanking regions of the breakpoint and are obtained by read pairs mapping at distances that exceed the library insert size distribution. When a low proportion of molecules carry the deletion, the probability to distinguish true signals from noise is low. In addition, PEM is best suited for relatively small variation (usually <50 kb). DOC methods can identify larger variation, but also suffer from a high noise‐to‐signal ratio, unless only very high deviations in coverage are considered. Detection of small fluctuations can be confounded by the presence of short strong variances generated by the presence of repetitive elements or other sequencing biases. In addition, DOC methods cannot detect balanced SV.

Single cell sequencing has been used to detect mosaic SV (McConnell *et al*., 2013), but this strategy requires to individually sequence a high number of cells. We reasoned that mosaic SV should result in a reduction of heterozygosity (ROH) that can be detected using heterozygous SNPs in NGS data. In heterozygous genomic positions, the two alleles are expected to be equifrequent (*p* = *q* = 0.5), and heterozygosity is 2*p*q = 0.5, which is the maximum theoretical value. When a mosaic SV occurs, the frequency of the two alleles varies, thus leading to a reduction of heterozygosity (ROH). We present χ‐scan, a software that addresses the need for a method specifically developed for mosaic SV detection that is more accurate and sensitive than standard approaches for SV detection and yet does not require single cell sequencing.

A schematic representation of deviations in relative allele frequency caused by different mosaic SV is provided in Figure 1. Χ‐scan uses information on SNP heterozygosity from two samples of somatic cells originating from a single zygote. These could be tumour and normal tissue from a patient, two plant clones obtained by vegetative propagation or cells from two different parts of the same plant. Details on the implementation of χ‐scan can be found in Methods S1, and a schematic representation and the pseudocode of the algorithm are presented in Figure S1 and Table S1, respectively. Briefly, the distortion of the relative allele frequency of heterozygous SNPs in one sample compared to the other is used as a proxy to detect a reduction of heterozygosity (ROH).

**Figure 1:**
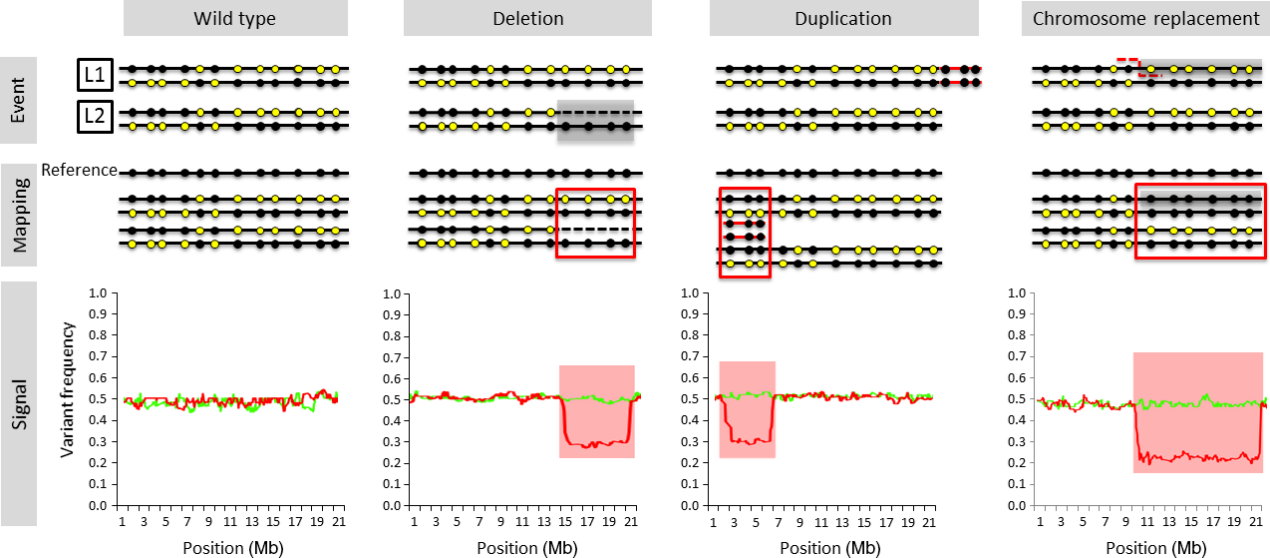
Deviations in allele frequency caused by several kinds of mosaic SV. Black and yellow dots represent the reference and alternative allele in heterozygous SNPs, respectively. The variant allele frequency is calculated as the proportion of reads carrying the variant allele. The graphs in the bottom line are the default graphs produced by χ‐scan. The green line represents variant allele frequency in the wild type clone and the red line represents variant allele frequency of the clone carrying the SV listed in the header (Wild type/No SV, Deletion, Duplication, and Chromosome replacement, respectively). Colored background indicates regions in which the deviation in allele frequency is statistically significant. [Correction added on 21 April 2017, after first online publication: The published figure 1 was previously incorrect and this has been amended in this version.]

We tested the ability of χ‐scan to identify simulated deletions occurring as mosaics in varying proportion of cells by computing F1 scores at decreasing relative abundance of reads carrying the variant allele and using different detection thresholds (Methods S1). The performance of χ‐scan was compared with that of other tools for the detection of SV: DNAcopy, BDmax, Control‐FREEC and DELLY. Χ‐scan showed significantly higher median F1 score than all other tools in almost all the simulated scenarios. Overall, the average F1 score achieved by χ‐scan across all simulated scenarios was 50%, compared to 29% achieved by DNAcopy, 41% by BDmax, 42% by DELLY and 16% by Control‐FREEC (Figure S2). Our results show that even SV observed in less than 10% of the reads (chromosomes) can be detected by χ‐scan (Figure S2).

Previous analyses have shown the mosaic nature of the mutation in Pinot gris that involves the L2 cell layer but not the L1 one (Pelsy *et al*., 2015; Vezzulli *et al*., 2012). Figure S3 shows the results of the identification of SV in chromosome 2 of Pinot gris (left) and Pinot blanc (right) with several tools leveraging NGS data. While all the methods identified the SV in Pinot blanc, χ‐scan was the only tested algorithm able to detect the SV in Pinot gris, identifying a strong ROH signal. Our results pinpointed the subcentromeric boundary of the SV at 13.6 Mbp and the telomeric boundary at 18.78 Mbp, at the end of chromosome 2.

To fully understand the nature of the identified SV, we integrated χ‐scan results with DOC information obtained using DNAcopy (Figure S4). We estimated the proportion of reads carrying the SV in Pinot gris to be 0.17, meaning that 66% of Pinot gris cells are carrying the variant allele, according to equation 1 in Methods S1. In spite of the high proportion of mutated chromosomes, there is no evidence of alteration in the coverage ratio of Pinot gris to both Pinot blanc and Pinot noir (top and centre plot, respectively) in the region affected by structural variation in Pinot gris (blue box in Figure S4), suggesting a balanced nature of the SV. On the contrary, the coverage of Pinot blanc showed a decrease in the region affected by the Pinot blanc deletion, relative to Pinot gris and Pinot noir (red box, centre and bottom plot, respectively), confirming the unbalanced nature of the SV. These results challenge the former interpretation that the observed large SV in Pinot gris is due to a deletion (Vezzulli *et al*., 2012), and confirm the more recent hypothesis of the variation being a chromosomal replacement (Pelsy *et al*., 2015). This might be the consequence of a double‐strand break, caused by DNA repair via break‐induced replication (BIR) (Llorente *et al*., 2008) or of a mitotic crossing over (Lee *et al*., 2009). Replacement of one chromosome with its homolog in cells of the L2 cell layer would explain why coverage does not decrease while allele frequency of SNPs is altered in leaf DNA.

The major limitation of χ‐scan in the detection of unbalanced SV is that it gives no information on the direction of the variation. However, this information can be determined by integrating coverage data with χ‐scan results. χ‐scan is the first software specifically developed for the detection of mosaic SV and can detect SV that do not involve copy number variation, such as mitotic crossing over or break‐induced recombination.

χ‐scan will help the identification of mosaic structural variation associated with novel phenotypic variation in several crop species, thus enhancing marker assisted breeding, and will be of help in the identification of genetic variation in clonally propagated crop species. χ‐scan is freely available at https://bitbucket.org/dscaglione/xscan/overview.

## Supporting information


**Figure S1** Representation of the algorithm implemented in χ‐scan.
**Figure S2** Performance of several tools in the detection of mosaic SV.
**Figure S3** Structural variation detected in V. vinifera chromosome 2 by different methods.
**Figure S4** Depth of Coverage analysis.
**Table S1** Simplified pseudocode to summarize the main steps of the algorithms.
**Methods S1** Supplementary methods.
