# Supplementary material for: Reduction of heterozygosity (ROH) as a method to detect mosaic structural variation
Source: Plant Biotechnol J. 2017 Mar 16;15(7):791–3. doi: 10.1111/pbi.12691 (PMC5466433; doi:10.1111/pbi.12691)
Supplement: Supplementary file 1 — Figure S1 Representation of the algorithm implemented in χ‐scan. Figure S2 Performance of several tools in the detection of mosaic SV. Figure S3 Structural variation detected in V. vinifera chromosome 2 by different methods. Figure S4 Depth of Coverage analysis. Table S1 Simplified pseudocode to summarize the main steps of the algorithms. Methods S1 Supplementary methods. [file PBI-15-791-s001.docx]

# Reduction of heterozygosity (ROH) as a method to detect mosaic structural variation.

## Supplementary Methods

**Sequencing**

Grapevine leaves for DNA extraction were sampled from stocks of certified clones, held at Vivai Cooperativi Rauscedo, Italy. The certified clones were Pinot blanc VCR5, Pinot gris R6 and Pinot noir VCR18 and Pinot Meunier SMA829.

DNA was extracted from nuclei and fragmented with Bioruptor® (Diagenode s.a., Seraing, Belgium) according to standard procedures (3 cycles of 15’’ ON and 90’’ OFF).

For Pinot blanc and Pinot Meunier, DNA was size selected by gel electrophoresis in the intervals of 400-700 bp. For each clone, two libraries with fragments of different size were prepared separately, according to Illumina TruSeq™ DNA protocol (Illumina, San Diego, CA). Pinot gris and pinot noir DNA libraries were prepared according to the Nextera DNA Sample Preparation Kit protocol (Illumina, San Diego, CA) with a resulting insert size of 400-900bp. 100bp-paired end reads were obtained with an Illumina Hiseq2000 from each library. All the above mentioned reads are archived on SRA, under Bioproject PRJNA321480.

**Alignment and SNP calling**

Raw fastq files generated by Illumina Hiseq2000 (Illumina, San Diego, CA) sequencers were trimmed by quality using the package ERNE-filter (Vezzi *et al.*, 2012). Trimmed reads longer than 50bp were retained for analysis. Adapter sequences were removed using cutadapt (Martin, 2011) and plastidial and mitochondrial reads were removed using ERNE-filter.

Short reads were aligned to the reference genome using the short read aligner BWA (Li and Durbin, 2009). The software package GATK (DePristo *et al.*, 2011) was used for local realignment of reads spanning small indels (tools RealignerTargetCreator and IndelRealignment), and for SNP calling (UnifiedGenotyper).

**χ-scan: problem definition and algorithm description**

The aim of χ-scan is to solve a structural variant detection problem in the context of somatic mosaicism. Let us characterize a generic region of the genome in terms of expected percentage of reads carrying a given allele. Normally, heterozygous variants (i.e. SNPs) would be expected to be found in 50% of the template DNA and we can talk of a σ-region with σ =0.5. In practice, the σ-value can vary for several reasons: i) alignment bias, ii) representation bias caused by PCR during library amplification, iii) GC content or iv) repetitive elements. Therefore, any σ-normal region should have σ = 0.5 ± ν^glob^, where ν^glob^ represents the constitutive deviation due to the aforementioned biases. Such variations are expected to be reproducible for the same σ region among different experiments with the same two haplotypes. Additional sources of variation are introduced by experimental measurements (e.g. probability distribution of allele sampling) and this will be denoted as ν^loc^. Hence, a σ-region not carrying any structural variation has σ = 0.5 ± ν^glob^ ± ν^loc^.

In the case of a deletion the σ-region has σ = 0.5 ± ν^glob^ ± ν^loc^ - μ, where μ measures the fraction of deleted allele molecules. A *mosaic variant* can be defined as a σ-region where μ is greater that ν^loc^ and less than 0.5 - ν^glob^ - ν^loc^, making σ greater than zero. The most delicate cases are those in which μ drops below ν^glob^ still being above ν^loc^ . In these cases, the possibility of detecting the variant rests on the assumption that the same ν^glob^ occurs both in test and control samples, and the rejection of null hypothesis is given by μ > ν^loc^.

χ-scan uses high coverage data (preferably 30x or more) to detect genomic regions characterized by differences in alternative allele frequency between two cell populations derived from the same zygote. Either cell population might harbor mosaic SVs. Deviations in allele frequency are calculated across windows of variable size, defined by a constant number of SNPs. Once the genome is segmented in overlapping windows, a statistical test is invoked to test for differences in the distribution of alternative allele frequency between the two samples for each window (i.e. testing if μ > ν^loc^). The implementation of χ-scan allows the use of two different approaches. The first, named “phased_fisher”, is meant to be a general-purpose system for heterozygous genomes, and is the one for which results are shown. The alternative algorithm, named “chi_reads”, has been implemented to leverage a higher statistical power when the haplotype phasing information is available for large segments of the zygote under analysis. This is the case of our analysis, since the reference genome of *Vitis vinifera* for most of its length consists of the same phases of any Pinot clone, being only interrupted by crossing-overs which occurred during self-pollination, used to obtain the near homozygous genotype used for assembly (Jaillon *et al.*, 2007). For such reason, one will expect to have (in crossing-over free windows) all reference (REF) nucleotides to derive from one chromosome, while alternative ones (ALT) derive from its counterpart.

The first part of the algorithm (Table S1) includes the acquisition of SNP information from a VCF file containing allele depth fields for each sample; when using the “phased_fisher” method, phased haplotype blocks are passed to the data structure. During the segmentation step, for each window under analysis (W), we define the vector P = (p_1_, p_2_, …, p_n_), where p_i_ is the position of the *i^th^* SNP retained in a given window. We describe the genotypic state of the test sample with the vectors t^ALT^ and t^REF^, and the genotypic state of the control sample with the vectors c^ALT^ and c^REF^. Each vector has length n, defined by the fixed number of SNP sites in the window. For each position *i*, c^REF^*_i_* and t^REF^*_i_* collect the number of reads carrying the reference allele, respectively for the control (c) and test (t) sample, while c^ALT^*_i_* and t^ALT^*_i_* collect the number of reads carrying the alternate allele. In the simplified approach (implemented in the “chi_reads” module), a chi-square test is applied to the sum of each vector along each window, as follows:

$T^{ALT}=\sum_{i=1}^{n} t_{i}^{ALT}$; $C^{ALT}=\sum_{i=1}^{n} c_{i}^{ALT}$ ; $T^{REF}=\sum_{i=1}^{n} t_{i}^{REF}$;$C^{REF}=\sum_{i=1}^{n} c_{i}^{REF}$

defining the following contingency table:

$$\begin{matrix} T^{\mathrm{ALT}} & T^{\mathrm{REF}} & T^{\mathrm{TOT}} \\ C^{\mathrm{ALT}} & C^{\mathrm{REF}} & C^{\mathrm{TOT}} \\ {ALT}^{\mathrm{TOT}} & {REF}^{\mathrm{TOT}} & \mathrm{TOTAL} \end{matrix}$$

Recalling the assumption that the Test and the Control originate from the same meiotic event (or, equivalently, from the same fertilized egg), in absence of mosaic structural variants the expectation of read count (E) for any given cell is equal to row total (R) * column total (C) / total (T). Deviations from the expected are tested performing a χ-square test on the contingency table, which in fact tests for μ > ν^loc^.

$$\chi^{2}=\sum_{i=1}^{R} \sum_{j=1}^{C} \frac{{(O_{ij}-E_{ij})}^{2}}{E_{ij}}$$

False Discovery Rate is then calculated by applying multiple test correction by the Benjamini-Hochberg’s method (Benjamini and Hochberg, 1995).

To generalize the method while maintaining multiple SNP site statistical power in the context where the reference genome does not represent one of the two haplotypes, we implemented a segmentation routine, which relies on previously computed haplotype blocks, reconstructed using HapCUT (Bansal and Bafna, 2008). A schematic representation of this approach is depicted in Figure S1. Each block generates a vector B = (b_1_, b_2_, …, b_n_) of phase statuses b, where n is the n*^th^* nucleotide in the haplotype block and b = {0,1}, and P’ = (p’_1_, p’_2_,…,p’*_i_* ) the genomic coordinate of a given position *i* along the chromosome; b is defining to which of the two homologous chromosomes the reference allele belongs. For any window (W), we define a vector of window-blocks WB = (wb_1_, wb_2_, …, wb_k_) for the k phase blocks reconstructed by HapCUT, if $\left| \text{ P’ ∩ P} \right|\text{ }\text{> s}$, being s the minimum amount of SNPs required to carry contiguous phase information and wb_n_ $\subseteq$ W. For each wb_k_, we generate the sets of phased allelic counts c^0^_k,_ t^0^_k,_ t^1^_k_, t^1^_k_ where

c^0^_k_ = {c^REF^_i_ | b_i_ = 0 } $\cup$ {c^ALT^_i_ | b_i_ = 1 }

c^1^_k_ = {c^REF^_i_ | b_i_ = 1 } $\cup$ {c^ALT^_i_ | b_i_ = 0 }

t^0^_k_ = {t^REF^_i_ | b_i_ = 0 } $\cup$ {t^ALT^_i_ | b_i_ = 1 }

t^1^_k_ = {t^REF^_i_ | b_i_ = 1 } $\cup$ {t^ALT^_i_ | b_i_ = 0 }

and therefore partitioning the real haplotype counts within each window-block. A two-tailed Fisher’s exact test is then applied to each pw_k_ following the contingency table:

$$\begin{matrix} \text{c}_{\text{k}}^{\text{0}} & \text{t}_{\text{k}}^{\text{0}} \\ \text{c}_{\text{k}}^{\text{1}} & \text{t}_{\text{k}}^{\text{1}} \end{matrix}$$

Each window-block wb will be tested locally for μ > ν^loc^, relying on some available phased SNP sites. To leverage the statistical power across several window-blocks within a given window, we applied the Stouffer’s method of meta-analysis of p-values (Stouffer *et al.*, 1949). Each p-value from Fisher’s exact test is converted to a Z score, populating the vector Z for a given window W, where Z = (z_1_, z_2_, …, z_k_) by the k*^th^* block. A meta Z-score (Zm) is calculated following the weighted Stouffer’s formula:

$$Z_{m}\text{ \textasciitilde}\frac{\sum_{\text{i=1}}^{\text{k}} {\text{w}_{\text{i}}\text{Z}}_{\text{i}}}{\sqrt{\sum_{\text{i=1}}^{\text{k}} \text{w}_{\text{i}}^{\text{2}}}}$$

where *w* is the length of the *k^th^* window-block and Zi its relative Z-score. Z_m_ is then converted to a window-wise p-value. Once all windows’ p-values are calculated, the Benjamini-Hochberg correction is applied. Windows with statistically significant p-values are considered positive and contiguous positive windows (parameterized in software) define ranges where allelic imbalance is likely to occur. Ancillary routines of the software permit the joining of positive ranges by filling gaps due to some negative windows. In addition, refinement of range boundaries can be obtained by analyzing p-value fluctuation on range borders.

**χ-scan: implementation**

The software is written in Python language. The minimal input data is a multi-sample VCF file containing SNP calling for both samples under analysis (i.e. test and control). AD field must be present in sample information to retrieve allele-specific read counts. Given any two populations of cells originated from the same fertilized egg, the occurrence of a mosaic SV in one of them will cause a bias in allele frequency, compared to the wild type population. Optionally, but required if reference sequence is not expected to represent long haplotype blocks of the genome under analysis, user can provide phase data generated by HAPCUT (Bansal and Bafna, 2008). χ-scan comes with an utility script and executables which perform an automatic workflow to produce data phased by HAPCUT. Required inputs for this subroutine are the BAM alignment file from the control sample and the reference sequence in FASTA format as used for the alignment. χ-scan can utilize two statistical methods: chi_reads and phased_fisher. The first one does not require the HAPCUT file, while the second does.

The user has the option of specifying several parameters, among which:

**--min-sample-cov**: minimum coverage each sample must have to consider SNP site for window population

**--cov-ratio**: maximum fold-increase with respect to median coverage to retain a SNP site

**--min-one-het**: min allele frequency at least one sample must have to retain SNP site

**--min-wins**: minimum number of widows to define a positive range

**--min-phased-collect**: Minimum number of phased SNP to retain a block from HAPCUT file; this will defined the set of sampled SNPs for windows generation

**--blocks-thresholds**: the following three numbers, space separated

- **min_block_size**: minimum number of SNP in a block to be fed into meta-analysis
- **min_block_num**: minimum number of window-blocks to trigger meta-analysis
- **min_SNP_perwin**: min amount of SNPs in window after block filtering (by size and numbers)

--**SNPwin**: Number of SNPs per window during VCF and HAPCUT parsing (deafult: 200)

--**overlap**: Number of overlapping SNPs between windows (deafult: 180)

--**no-gapfill**: Skip gap filling routine

--**refine**: Perform refinement of ranges by inspection of pvalue slopes (default: disabled)

--**max-gap**: (Gapfill phase 1): Gap between positive windows will be filled if shorter than max-gap

--**gap-ratio**: (Gapfill phase 2): Max ratio between gap and shorter flanking positive range; if a gap is present between two positive ranges and such gap is shorter that max gap, the gap is filled to build a single range.

--**method**: {chi_reads, phased_fisher}

--**select-refs [CONTIGS [CONTIGS ...]]**: Limit to a set of reference sequences to operate on (space separated)

--**min-adj-pvalue**: Minimum Benjamini-Hochberg adjusted p-value to consider positive windows

Full documentation is provided at <https://bitbucket.org/dscaglione/xscan/overview>.

The use of highly overlapped sliding windows is recommended to precisely define the borders of SVs, as the increase in computation time is small compared to the parsing time. In the present study, analyses were performed on overlapped sliding windows of size 200 SNPs and step 20 SNPs.

**Generation and analysis of simulated samples**

The *V. vinifera* reference genome was assembled from the highly homozygous line PN40024 resulting from several cycles of selfing of a Pinot noir x Helfesteiner cross (Jaillon *et al.*, 2007). As a result, Pinot clones are expected to differ from PN40024 for at most one allele across large chromosome blocks. We used the GATK FastaAlternateReferenceMaker (DePristo *et al.*, 2011) tool to obtain a reference carrying the alternative alleles in heterozygous sites obtained from resequencing data of Pinot blanc and Pinot Meunier. We will refer to the two references as PN (PN40024) and PA (Pinot Alternative), respectively.

A set of 50 deletions was simulated in each reference. Size of the simulated deletions ranged from 107 Kbp to 1.67 Mbp. The position of each deletion in the genome was randomly chosen with the only constraint that deletions do not overlap. We will refer to deleted reference sequences as dPN and dPA respectively.

Fifty million reads were generated from each of the four references: PN40024 haplotype (PN), PN40024 alternative haplotype (PA), PN40024 haplotype carrying deletions (dPN) and PN40024 alternative haplotype carrying deletions (dPA). Paired reads were simulated using wgsim (https://github.com/lh3/wgsim/), setting read length of both reads of a pair to 100 and genotyping error to 1%.

Simulated reads were then mixed in variable proportions (for a total of 100 million read pairs) to simulate the chimeric deletions in the two references as follows:

1. Samples carrying deletions at different frequencies in the PN haplotype were simulated by mixing a proportion of 0.5 reads simulated from PA, a proportion of x reads simulated from dPN and a proportion of y of reads simulated from PN; where x=0.05, 0.75, 0.1 or 0.2, while y is given by 0.5-x.
2. Samples carrying deletions at different frequencies in the PA haplotype were simulated by mixing a proportion of 0.5 of reads simulated from PN, a proportion of x reads simulated from dPA and a proportion of y reads simulated from PA; where x=0.05, 0.75, 0.1, 0.2, while y is given by 0.5-x.

This allowed the creation of samples carrying a proportion of deleted haplotype of 5%, 7.5%, 10%, and 20% respectively.

To facilitate downstream analysis, the comparisons were carried out between samples carrying deletions with the same frequencies in the two different haplotypes, i.e. the sample carrying deletions simulated with a frequency of 5% in the PN haplotype was compared with the sample carrying deletions simulated with a frequency of 5% in the PA haplotype.

For each of the utilized methods, the choice of the threshold discriminating between positives and negatives might affect performance. Simulated results were thus analyzed by varying the detection threshold. For each approach we defined a permissive threshold and a conservative threshold, and divided the space between the two in 25 equally spaced intervals, to assess global performance at varying thresholds.

The threshold for χ-scan is based on adjusted p-value for the existence of significant differences in allele frequencies between two samples. We chose 0.1 as a permissive threshold and 10^-4^ as a restrictive threshold. To avoid to excessively sample near the permissive threshold, the space between log10(0.1) and log10(10^-10^) was divided into 25 equally spaced intervals.

The threshold for DNAcopy (Olshen *et al.*, 2004) and control-freec (Boeva *et al.*, 2012) is the absolute value of the log2ratio of the coverage of the two samples in any given region. We chose 0 as a permissive threshold and 0.5 (slightly greater than the expected log2ratio in case of 20% deleted reads) as a conservative threshold.

The threshold used for BreakDancerMax (BDmax) (Chen *et al.*, 2009) and DELLY (Rausch *et al.*, 2012) was the number of reads supporting a deletion. The permissive threshold was set at 2 and the restrictive threshold was set at 25.

The proportion of reads originated from the deleted reference sequence in the simulation represents an approximation of the proportion of cells carrying a deletion in a heterogeneous population of cells.

Performance of each software tool was assessed using the F1 score. The F1 score is calculated as 2*precision*recall/(precision+recall), equivalent to 2*TP/(2*TP+FP+FN) (Sokolova *et al.*, 2006). The F1 statistics is preferred to accuracy, defined as (TP+TN)/(TP+TN+FP+FN), when a large part of results is expected in the negative category, as in the present analysis.

Assuming that in wild-type samples the two alleles of a heterozygous SNP are equifrequent, the proportion of cells carrying the variant in mosaic tissues (*PVC*) can be estimated as follows:

1. When the mutation does not change copy number (e.g. chromosome replacement)

$PVC=\left| 1-\frac{F_{mut}}{F_{wt}} \right|$ (1)

1. When the mutation is a deletion

$PVC=2*\frac{\left( F_{wt}-F_{mut} \right)}{(2*{F_{wt}-F}_{mut})}$(2)

where Fmut is the frequency of the allele on the mutated chromosome in the mosaic sample and Fwt is the frequency of the same allele in the wild-type sample, respectively.

**Detection of SVs with available tools**

*Depth Of Coverage*

DNAcopy (Olshen *et al.*, 2004) was originally developed for the detection of copy number variants in comparative genomic hybridization (CGH) experiments, but has been applied to the detection of somatic CNVs in cancer, leveraging NGS data (Zack *et al.*, 2013). To fully exploit DNAcopy capabilities, the reference genome was segmented in windows of variable size and containing a constant number of mapped reads. The length of the windows was calculated by simulating short reads from the reference and mapping them back to the reference. This avoided the creation of spurious copy number signals due to windows in which only a very small number of reads mapped.

The log2 ratio of the coverage of each window was computed between the two studied samples and the circular binary segmentation algorithm implemented in DNAcopy was used to identify regions of copy number variation.

Control-FREEC (Boeva *et al.*, 2012) is a tool developed for estimating copy number alterations and allelic imbalances in tumors. Control-FREEC identifies copy number alterations of a sample, optionally compared to a control, and can use information on beta allele frequency (BAF) profiles to improve the identification of structural variants. In the present study we used information on BAF profiles. Log2 ratio of the coverage of the samples to be compared was computed across windows of 5000 bp.

*Paired end mapping*

BreakDancerMax (BDmax) (Chen *et al.*, 2009) uses signatures of paired reads mapping at unusually large distances to identify deletions in the sequenced sample compared to the reference. DELLY (Rausch *et al.*, 2012) uses paired end information together with split read mapping information to detect structural variants. Methods based on paired end mapping (PEM) do not compare one sample with another, but one sample to the reference sequence. When searching for deletions of a sample compared to another, PEM-based approaches were run on both samples and results were integrated. BreakDancerMax (BDmax) and DELLY return the identified deletion together with additional information, including the number of reads supporting each deletion. Only deletions supported by at least two reads were retained for analysis. In addition, DELLY provides a flag for low confidence calls and deletions flagged as low confidence were removed from further analysis.

## References

**Bansal, V. and Bafna, V.** (2008) HapCUT: an efficient and accurate algorithm for the haplotype assembly problem. *Bioinformatics*, **24**, i153–9.

**Benjamini, Y. and Hochberg, Y.** (1995) Controlling the False Discovery Rate: a Practical and Powerful Approach to Multiple Testing. *J. R. Stat. Soc. Ser. B*, **57**, 289–300.

**Boeva, V., Popova, T., Bleakley, K., Chiche, P., Cappo, J., Schleiermacher, G., Janoueix-Lerosey, I., Delattre, O. and Barillot, E.** (2012) Control-FREEC: a tool for assessing copy number and allelic content using next-generation sequencing data. *Bioinformatics*, **28**, 423–5.

**Chen, K., Wallis, J.W., McLellan, M.D., et al.** (2009) BreakDancer: an algorithm for high-resolution mapping of genomic structural variation. *Nat. Methods*, **6**, 677–81.

**DePristo, M.A., Banks, E., Poplin, R., et al.** (2011) A framework for variation discovery and genotyping using next-generation DNA sequencing data. *Nat. Genet.*, **43**, 491–8.

**Jaillon, O., Aury, J.-M., Noel, B., et al.** (2007) The grapevine genome sequence suggests ancestral hexaploidization in major angiosperm phyla. *Nature*, **449**, 463–7.

**Li, H. and Durbin, R.** (2009) Fast and accurate short read alignment with Burrows-Wheeler transform. *Bioinformatics*, **25**, 1754–60.

**Martin, M.** (2011) Cutadapt removes adapter sequences from high-throughput sequencing reads. *EMBnet.journal*, **17**, pp. 10–12.

**Olshen, A.B., Venkatraman, E.S., Lucito, R. and Wigler, M.** (2004) Circular binary segmentation for the analysis of array-based DNA copy number data. *Biostatistics*, **5**, 557–72.

**Rausch, T., Zichner, T., Schlattl, A., Stütz, A.M., Benes, V. and Korbel, J.O.** (2012) DELLY: structural variant discovery by integrated paired-end and split-read analysis. *Bioinformatics*, **28**, i333–i339.

**Sokolova, M., Japkowicz, N. and Szpakowicz, S.** (2006) *AI 2006: Advances in Artificial Intelligence* A. Sattar and B. Kang, eds., Berlin, Heidelberg: Springer Berlin Heidelberg.

**Stouffer, S.A., Suchman, E.A., Devinney, L.C., Star, S.A. and Williams, R.M.J.** (1949) *The American Soldier: Adjustment during army life*, Princeton, NJ: Princeton University Press.

**Vezzi, F., Fabbro, C. Del, Tomescu, A.I. and Policriti, A.** (2012) rNA: a fast and accurate short reads numerical aligner. *Bioinformatics*, **28**, 123–4.

**Zack, T.I., Schumacher, S.E., Carter, S.L., et al.** (2013) Pan-cancer patterns of somatic copy number alteration. *Nat. Genet.*, **45**, 1134–1140.

## Supplementary Tables

|  |  |  | |  |  | | | |  |  |  |  |  |  |  |  |
| --- | --- | --- | --- | --- | --- | --- | --- | --- | --- | --- | --- | --- | --- | --- | --- | --- |
| **Preconditions:** | | | |  |  | |  | | |  |  |  |  |  |  |  |
| Let SNPS be the array with length *n* of available SNP sites identified on a chromosome | | | | | | | | | | | | |  |  |  |  |
| Let PHASES be the array with length *k* of available haplotype blocks on a chromosome | | | | | | | | | | | | |  |  |  |  |
|  |  |  | |  |  | |  | | |  |  |  |  |  |  |  |
| **Initialization of SNPs and PHASEs** | | | | | | | | | | | | | |  |  |  |
| χ-scan (*k*,*n*)  **for** *i* ← 1 **to** *n* **do** | | | | | | | | |  |  |  |  |  |  |  |  |
|  | | | *data_SNP* ← snp_data_collection(*SNPS_i_*)  *# SNP_i_ is the i^th^SNP site described in a VCF file format produced by GATK* | | | | | | | | | | | |  |  |
| **for** *i* ← 1 **to** *k* **do** | | | | | | | | |  |  |  |  |  |  |  |  |
|  | | | *data_PHASES* ← phase_data_collection(*PHASES_i_*)  *# PHASES_i_ is the i^th^PHASE BLOCK described in a HAPCUT file format* | | | | | | | | | | | | |  |
| **Creation of WINDOWS based on number of SNPs** | | | | | | | | | | | | | |  |  |  |
|  | | |  | | | | | |  |  |  |  |  |  |  |  |
| *WINDOWS* ← define_windows(*data_SNP, data_PHASES*) | | | | | | | | | | | | | | | |  |
|  | | |  | | | | | |  |  |  |  |  |  |  |  |
| **Computation of test statistics and pvalues** | | | | | | | | | | | | | |  |  |  |
| for *win* in *WINDOWS*: | | | | | |  |  |  |  |  |  |  |  |  |  |  |
|  | | | *pvalues* ← test_window(*win*) | | | | |  |  |  |  |  |  |  |  |  |
|  | | | *fdr* ← correction(*pvalues*) | | | | |  |  |  |  |  |  |  |  |  |
|  | | |  | | | | | |  |  |  |  |  |  |  |  |
|  |  |  | |  |  | |  | | |  |  |  |  |  |  |  |

**Table S1: Simplified pseudocode to summarize the main steps of the algorithms.** The “phased_fisher” procedure is depicted here, acquiring phase information to generate windows.

## Supplementary Figures

**
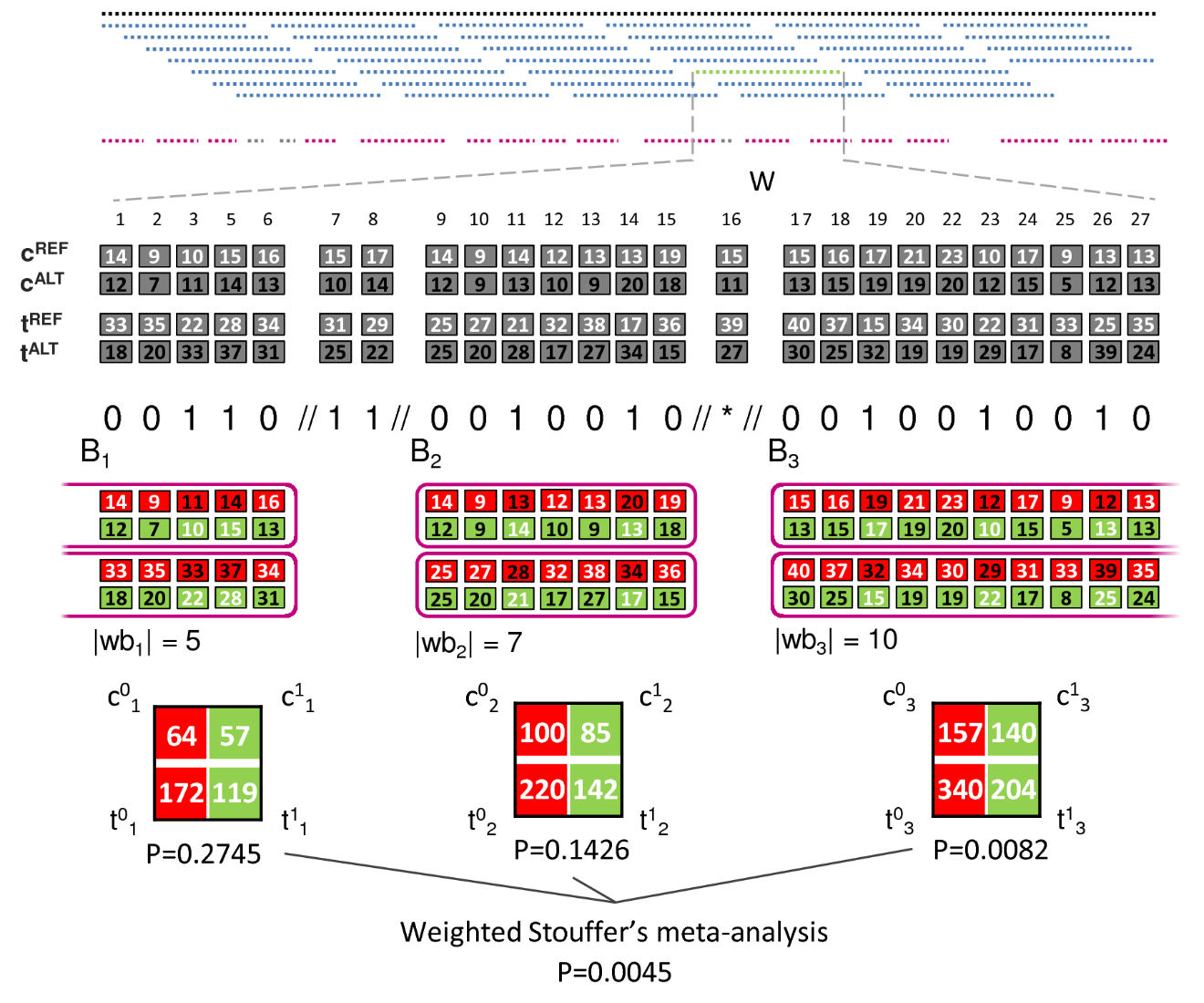
**

**Figure S1**: **Representation of the algorithm implemented in χ-scan.** The figure details the *phased_fisher* algorithm (see Supplementary Methods for details). Overlapping windows of SNPs (blue dotted lines) are generated from all the available heterozygous SNPs along the chromosome (black dotted line). Reads counting the reference allele are in bold white, while reads calling an alternative allele are in bold black. Reconstructed phase blocks (purple dotted lines) are acquired from HAPCUT algorithm and joined with the SNP data with respective allele counts (c^REF^, c^ALT^, t^REF^, t^ALT^ ) for each of the two samples. SNP data included in phase blocks and satisfying a minimum amount of phased SNPs, generate window-block objects (wb) using a switching mask (1/0) to represent the nucleotide counts residing on the same homologous chromosome. Phase interruption are represented by double slashes. The 7^th^ and the 8^th^ SNP sites are excluded as they belong to a phase block not satisfying the SNP number limit, while 16^th^ SNP is excluded from calculation as it does not retrieve any phase information. In-phase allelic counts are then summed to populate the contingency table at window-block level to perform a two-tailed Fisher’s exact test. P values are then meta-analyzed using a weighted Stouffer’s method, using as weight the amount of SNPs within each window-block.

**
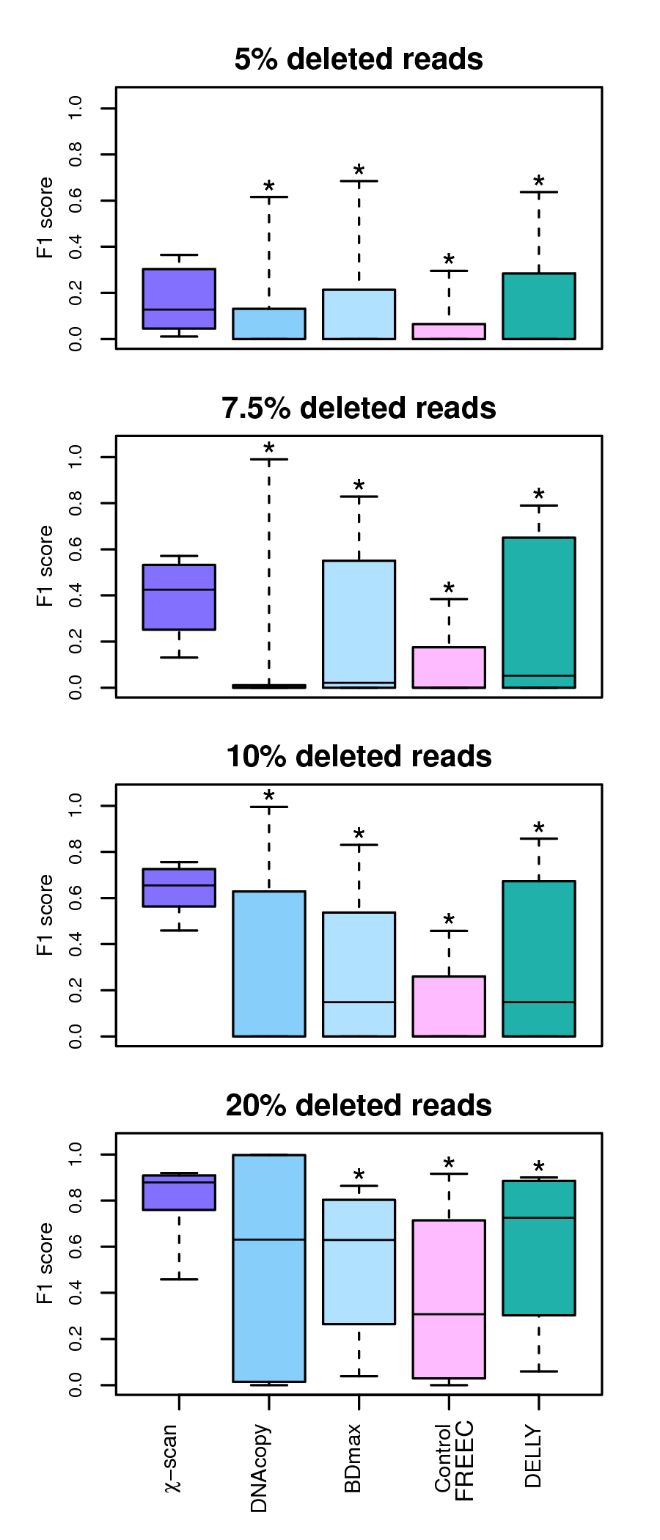
**

**Figure S2:** **Performance of several tools in the detection of mosaic deletions.** Performance is measured as F1 score and was measured using different amount of reads originated from deleted reference sequences (5%, 7.5%, 10% and 20%, top to bottom). *= F1 score of the tool significantly lower than χ-scan F1 score.

**
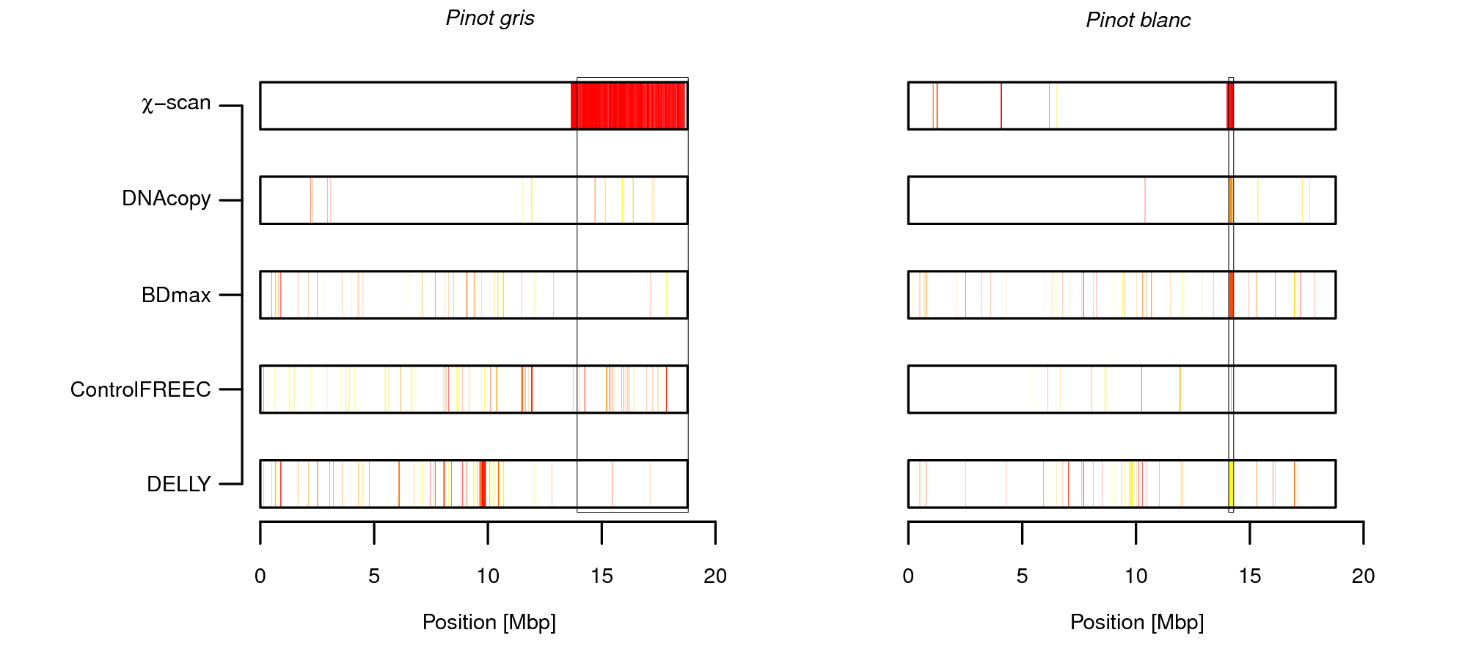
**

**Figure S3**: **Structural variants detected in *V. vinifera* chromosome 2 by different methods.** Identification of structural variants in Pinot gris (left) and in Pinot blanc (right). The black boxes delimit the borders of previously reported SVs. Colored regions denote a structural variant reported by the considered approach with colors indicating the strength of signal (white=no signal, red=strongest signal). Strength of the signal was measured as p-value for χ-scan, log2 ratio of coverage for DNAcopy and Control-FREEC, and number of supporting reads for BDmax and DELLY.

**
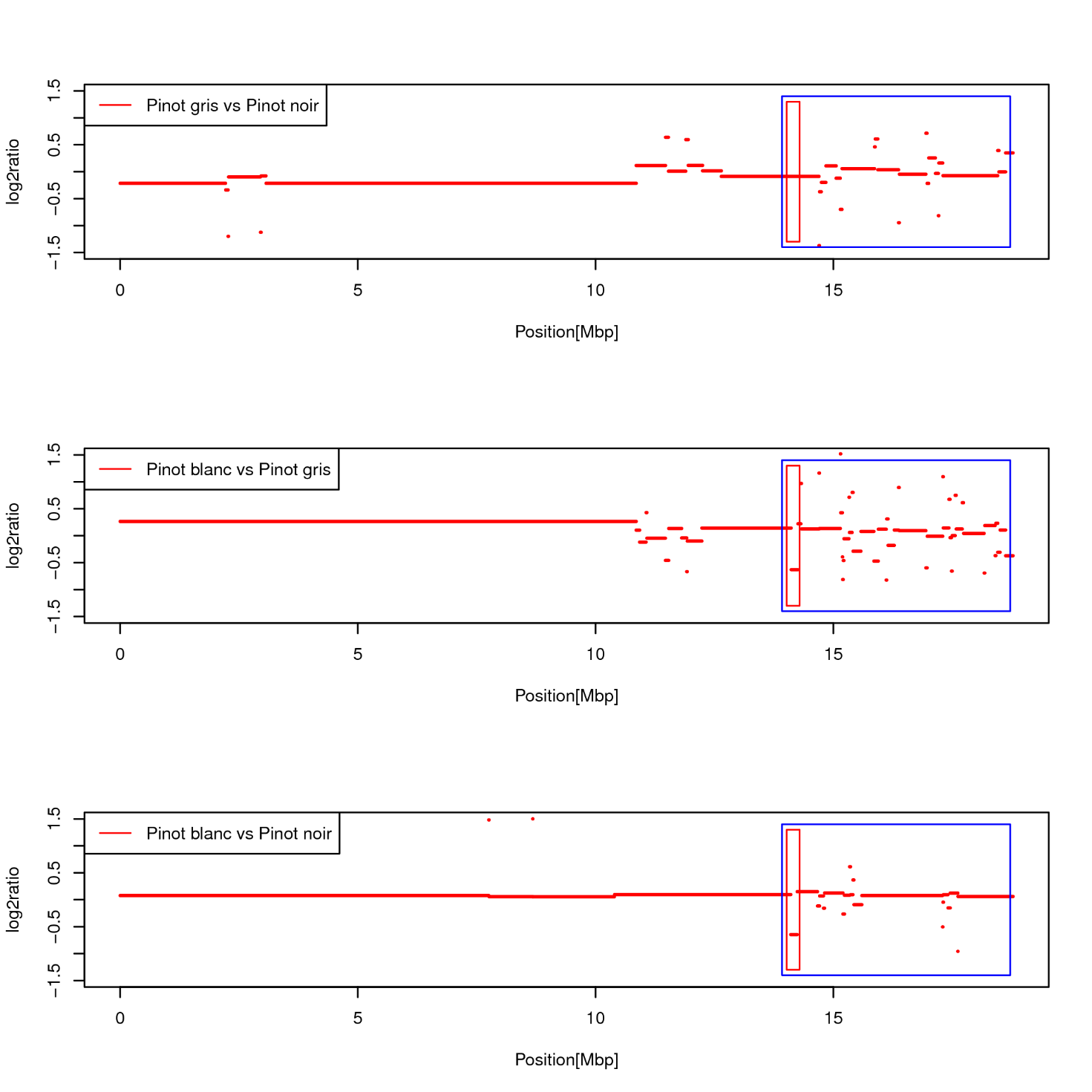
Figure S4:** **Depth of Coverage analysis.** Log2 ratio of chromosome 2 coverage in Pinot gris vs Pinot noir (top), Pinot blanc vs Pinot gris (center) and Pinot blanc vs Pinot noir (bottom). Blue vertical lines delimit the location of the SV region in Pinot gris. Red vertical lines delimit the location of the SV region in Pinot blanc.
